# Neutrophil extracellular traps contribute to the pathogenesis of acid-aspiration-induced ALI/ARDS

**DOI:** 10.18632/oncotarget.22744

**Published:** 2017-11-28

**Authors:** Haitao Li, Xiaoting Zhou, Hongyi Tan, Yongbin Hu, Lemeng Zhang, Shuai Liu, Minhui Dai, Yi Li, Qian Li, Zhi Mao, Pinhua Pan, Xiaoli Su, Chengpin Hu

**Affiliations:** ^1^ Department of Pulmonary and Critical Care Medicine, Key Site of National Clinical Research Centre for Respiratory Disease, Xiangya Hospital, Central South University, Changsha 410008, China; ^2^ Department of Neurology, Xiangya Hospital, Central South University, Changsha 410008, China; ^3^ Department of Respiratory Medicine, Changsha Central Hospital, Changsha, 410004, China; ^4^ Department of Pathological Medicine, Xiangya Hospital, Central South University, Changsha 410008, China; ^5^ Department of Thoracic Medicine, Hunan Cancer Hospital, Affiliated to Xiangya Medical School, Central South University, Changsha, 410013, China

**Keywords:** neutrophil extracellular traps(NETs), acid-aspiration, acute lung injury(ALI), acute respiratory distress syndrome(ARDS)

## Abstract

**Background:**

Acute lung injury/acute respiratory distress syndrome (ALI/ARDS) is a manifestation of systemic inflammation in the lungs, but the factors that trigger inflammation in ALI/ARDS are unclear. We hypothesized that neutrophil extracellular traps (NETs) contribute to the pathogenesis of acid aspiration-induced ALI/ARDS.

**Results:**

Analysis of bronchial aspirates from ARDS patients showed that NETs were significantly correlated with the degree of ARDS (r = –0.5846, *p* = 0.0359). NETs in bronchoalveolar lavage fluid of acid-aspiration mice were significantly higher (141.6 ± 23.08) at 3 h after injury than those in the sham group (1234 ± 101.9; *p* = 0.003, *n* = 5 per group). Exogenous NETs aggravated lung injury, while alvelestat and DNase markedly attenuated the intensity of ARDS.

**Materials and Methods:**

We investigated whether NETs are involved in the severity of gastric aspiration-induced ARDS. Then, a hydrochloric acid aspiration-induced ALI murine model was used to assess whether NETs are pathogenic and whether targeting NETs is protective. Exogenous NETs were administered to mice. Alvelestat can inhibit neutrophil elastase (NE), which serves an important role in NET formation, so we investigated whether alvelestat could protect against ALI in cell and mouse models.

**Conclusions:**

NETs may contribute to ALI/ARDS by promoting tissue damage and systemic inflammation. Targeting NETs by alvelestat may be a potential therapeutic strategy.

## INTRODUCTION

Acute lung injury/acute respiratory distress syndrome (ALI/ARDS) is caused by inflammatory lung injury that results in severe, diffuse increased alveolar-capillary permeability. Consequently, edema, hypoxia, and hemorrhage follow. Effective advanced care measures, such as lung-protective ventilation and fluid-conservation management, have been applied in clinical practice for years, but ARDS is still a major risk to patients in intensive care units, with hospital mortality rates of 40% or more [1, 2].

Pneumonia, aspiration and sepsis are the leading causes of ARDS; these conditions lead to tissue injury and inappropriate accumulation of inflammatory factors, along with leukocyte activation. These changes eventually activate inflammatory cascades, which lead to alveolar barrier disruption, resulting in severe conditions such as respiratory failure [1, 3]. Common pathological features of ARDs include rapid recruitment of leukocytes and release of proinflammatory cytokines, triggering systemic inflammation [1, 4]. However, how these factors “pull the trigger” in ARDS is still unclear. Identification of core factors that mediate inflammation in ARDS is critical.

Neutrophil extracellular traps (NETs) are networks of extracellular fibers, primarily composed of DNA, which are embedded with histones, myeloperoxidase (MPO) and neutrophil elastase (NE) and released from neutrophils to bind pathogens [5]. Recently, NETs have been shown to be potential mediators in noninfectious systemic inflammatory diseases, including sepsis, acute ischemia-reperfusion injuries of the liver, and transfusion-related or LPS-induced acute lung injury [6–9]. These findings indicate that NETs have many functions, including induction of alveolar-capillary barrier damage, platelet aggregation, and cytokine production, all of which are likely involved in the pathogenesis of ALI/ARDS.

Aspiration was identified a major cause of ALI/ARDS in previous studies [10–13], and acid aspiration is known as a neutrophil-dependent form of ALI/ARDS characterized by injury of both the alveolar epithelium and the capillary endothelium [14, 15]. Therefore, we hypothesized that NETs in aspiration play a significant role in the progression of ARDS. Furthermore, inhibition of NET production or degradation of NETs may be potential therapeutic approaches in acid-aspiration-induced ALI/ARDS.

To verify this hypothesis, we first enrolled 13 patients with ARDS caused by aspiration of gastric contents to analyze the correlation between the clinical severity and the level of NETs. Then, we established an acid aspiration murine model of acute lung injury, which simulates acute gastric aspiration injury in the clinic [3, 16], to investigate whether NETs released after lung injury have pathogenic roles. In addition, we assessed whether inhibitors of NET production have beneficial effects in the acute lung injury model to identify new strategies for treatment of ARDS. A previous study showed that NE played a central role in the formation of NETs, and NE is one component of NETs [17–19]. Alvelestat (AZD9668), an NE inhibitor, can prevent human NE-induced lung injury in mice and rats and reduce the inflammatory response [20]. Therefore, we sought to explore whether alvelestat has the potential to attenuate acid aspiration-induced lung injury through degradation and inhibition of NETs.

## RESULTS

### NETs indicate disease severity in gastric aspiration-induced ARDS patients

**Figure 1:**
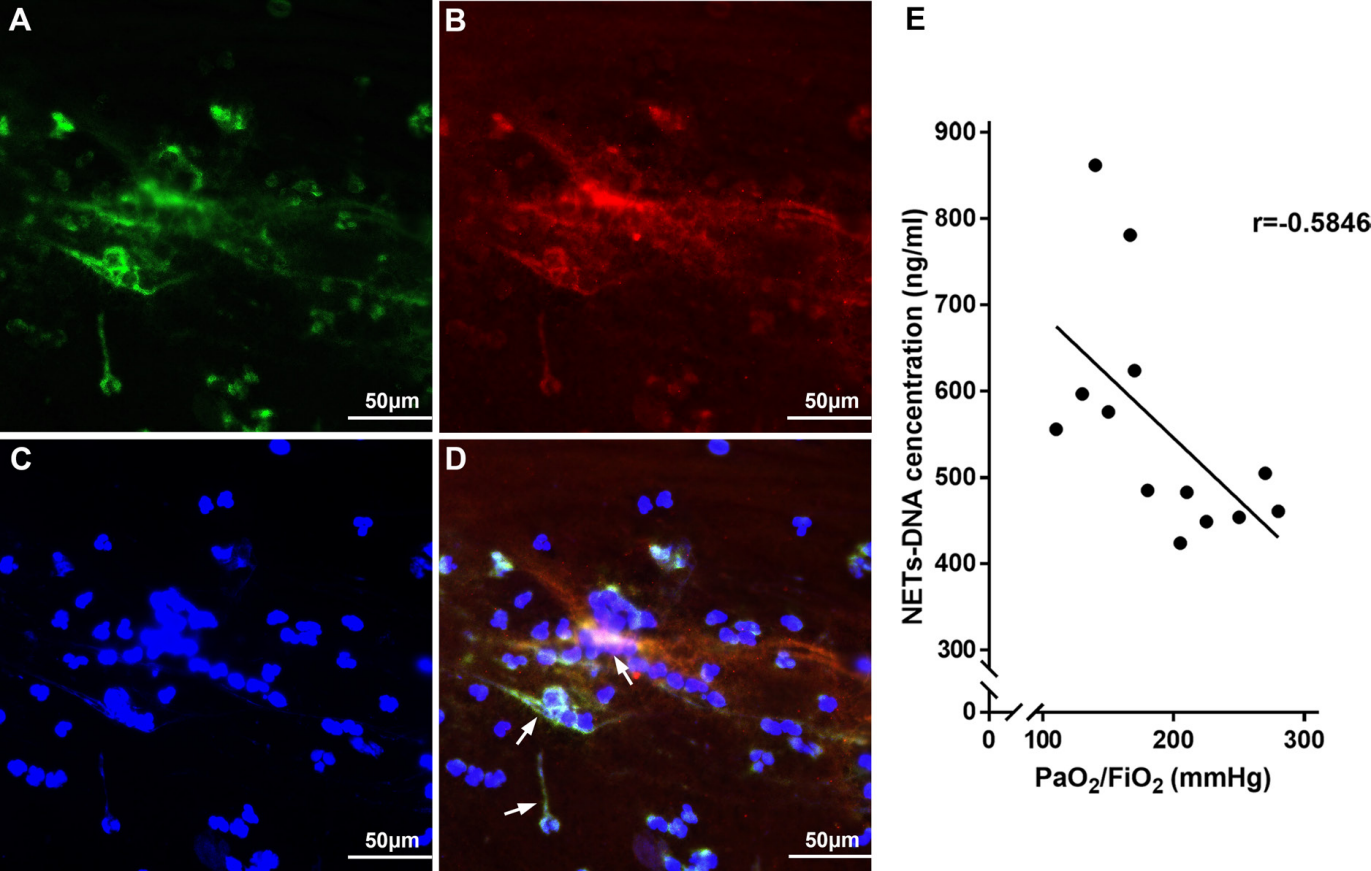
NETs were observed to determine the clinical relevance to disease severity in gastric aspiration-induced ARDS patients NETs (white arrow in **D**) were characterized with cit-H3 (**A**), NE (**B**) and DNA (**C**) in bronchial aspirates from gastric aspiration-induced ARDS patients. (**E**) PaO_2_/FiO_2_ is highly correlated to NET concentration in bronchial aspirate from gastric aspiration-induced ARDS patients.

Thirteen ARDS patients (8 men and 5 women with a mean age of 54 ± 6.3 years) with a recent gastric aspiration history were enrolled in the study. All patients had a history of gastric aspiration events within one week, with associated aspiration signs, such shortness of breath and appearance of a new infiltrative shadow on a chest X-ray. We characterized NETs (Figure 1D) in bronchial aspirates by co-localization of citrullinated-histone3 (cit-H3) (Figure 1A) and NE (Figure 1B) using immunofluorescence analyses. Furthermore, we measured the NET-DNA levels in the supernatant of bronchial aspirates for quantitative analysis of these patients (Figure 1E). There was a significant correlation of NET-DNA in bronchial aspirate with arterial oxygen tension/inspired oxygen fraction (PaO_2_/FiO_2_) (r = –0.5846, *p* = 0.0359) in patients’ blood gas analysis index, suggesting that NETs aggravated pulmonary ventilation dysfunction in ARDS. We concluded that NETs might reflect disease severity of ARDS in clinical situations.

### NETs are increased in mice with acid aspiration-induced lung injury

**Figure 2:**
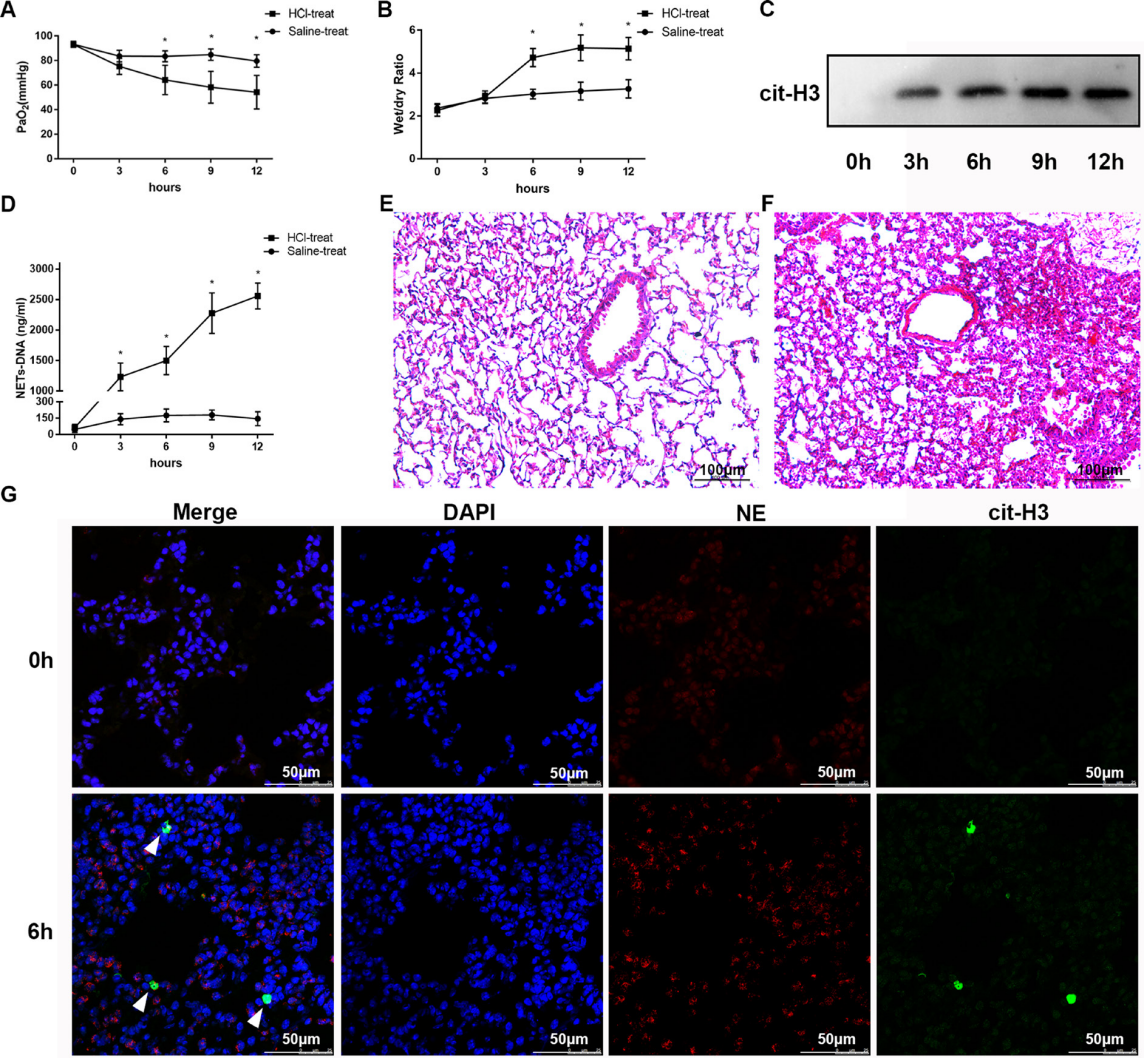
HCl aspiration caused ARDS as well as elevated NET levels in the BALF of mice (**A**) HCl was used to challenge the mice and resulted in a decrease in PaO_2_ in a time-dependent manner (^*^p < 0.05 vs. the sham group). (**B**) Mouse lung wet–dry ratios were augmented after HCl aspiration (^*^p < 0.05 vs the sham group). (**C**) An example of cit-H3 detected by Western blot in the BALF of mice after HCl aspiration. The blots are representative of at least three independent experiments. (**D**) HCl aspiration caused a significant increase in BALF NET-DNA of mice measured using PicoGreen (^*^p < 0.05 versus the sham group). Hematoxylin and eosin–stained sections of lung injury at 0 h (**E**) and 6 h (**F**) after HCl administration to mice. Obvious pathological changes were observed in the lungs of HCl-treated mice, such as alveolar hemorrhage and infiltration of neutrophils. Scale bars: 100 μm. (**G**) Confocal microscopy of lung sections with immunofluorescence at 0 h and 6 h after HCl administration to mice. The complex of cit-H3 and NE (NETs) substantially increased at 6 h, along with neutrophils. Scale bars: 50 μm.

Administration of HCl to mice resulted in significant lung damage, as reflected by the decreased PaO_2_ (83.4 ± 1.965 vs 64.2 ± 5.342, *n* = 5 per group, *p* = 0.0194; Figure 2A) and the increased lung wet/dry weight ratio (3.02 ± 0.102 vs 4.72 ± 0.1881 at 6 h, *n* = 5 per group, *p* = 0.0002; Figure 2B), both of which are indicators of ARDS. Lung histological examination at 6 h (Figure 2F) after HCl challenge demonstrated multifocal alveolar hemorrhage, diffuse disruption of the alveolar wall, and massive inflammatory cell infiltration, while at 0 h (Figure 2E), there was minimal lung injury. Immunofluorescence microscopy of lung tissue at 6 h after HCl challenge also showed the presence of NETs, as determined by co-localization of cit-H3 and NE (Figure 2G). Similarly, the levels of NET-DNA were increased in the BALF of mice after the HCl challenge (Figure 2D). As a semi-quantitative analysis of NETs, the presence of cit-H3 in BALF detected by Western blotting (Figure 2C) was increased in a time-dependent manner, with similar results obtained from lung tissue immunofluorescence of NETs. The release of NET-DNA into the BALF occurred in a time-dependent manner, which correlated with the severity of ARDS.

### Exogenous NETs aggravate lung inflammation in mice with ARDS

**Figure 3:**
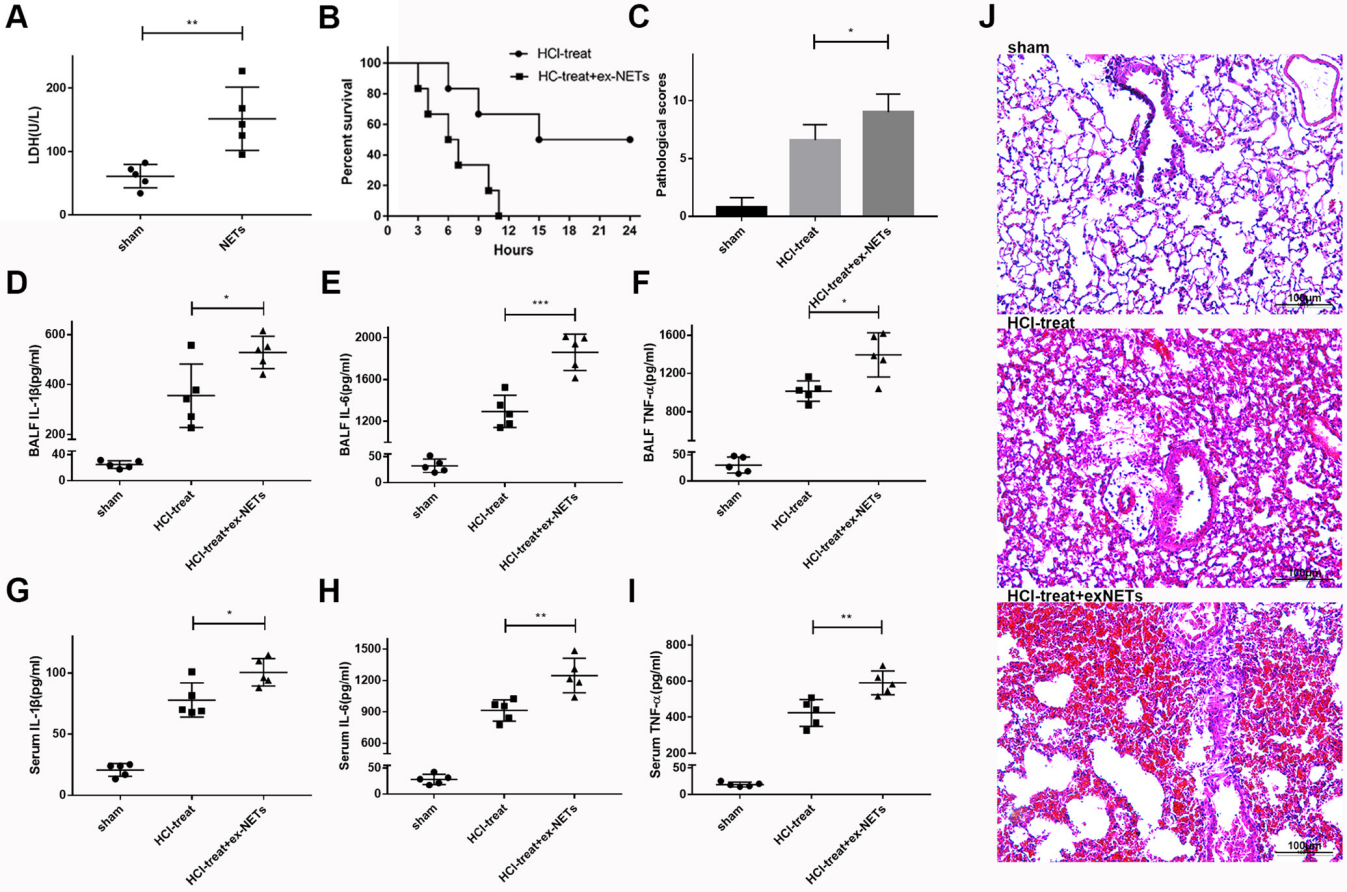
Exogenous NETs aggravated lung damage and inflammation in mice with ARDS (**A**) Pulmonary instillation of exogenous NETs (1.5 mg/kg) caused mild lung damage in mice, as evidenced by elevated BALF LDH levels at 12 h, compared to sham-treated mice. (**B**) NETs plus HCl significantly decreased the survival rate of HCl-treated mice. (**C**) Lung histological scores were higher in the NETs plus HCl groups than in the HCl group. BALF (**D**) IL-1β, (**E**) IL-6 (**F**) and TNF-α levels were all notably increased in the NETs plus HCl groups compared with HCl-treated mice, as were the serum (**G**) IL-1β, (**H**) IL-6 (I) and TNF-α levels. (**J**) Hematoxylin and eosin–stained sections of lung damage in the saline-treated group and NETs plus HCl groups compared to the HCl alone group. Scale bars: 100 μm. (^*^p < 0.05, ^**^p < 0.01, ^***^p < 0.001).

In further experiments, we assessed whether NETs released during ARDS contributed to inflammation and lung injury in this model. First, we showed that pulmonary instillation of mouse marrow-derived NETs at a DNA concentration of 1.5 mg/kg alone resulted in mild lung damage in mice, as shown by an increase in BALF LDH levels at 12 h, compared to saline-treated mice (61 ± 8.258 vs. 151.6 ± 22.29, *n* = 5 per group, *p* = 0.0052; Figure 3 A). Notably, administration of exogenous NETs immediately after HCl challenge significantly increased the death rate of mice compared with the HCl only-treated group (Figure 3B, *p* = 0.0209). Exogenous NETs aggravated acid-induced lung injury in mice, as shown by enhanced lung injury scores (HCl-treated group 6.6 ± 0.6 vs HCl-treated + exogenous NETs group 9 ± 0.7071, *n* = 5 per group, *p* = 0.0322; Figure 3C, 3J) and the increased inflammatory cytokines (TNF-α, IL-1β, IL-6) in BALF (Figure 3D–3F) and in plasma (Figure 3J–3I). Several of these cytokines are associated with the development and progression of ARDS in humans [21, 22]. Therefore, our data confirmed that NETs contribute to ARDS in mice after acid aspiration.

### Attenuation of NETs is protective in mice with lung injury

**Figure 4:**
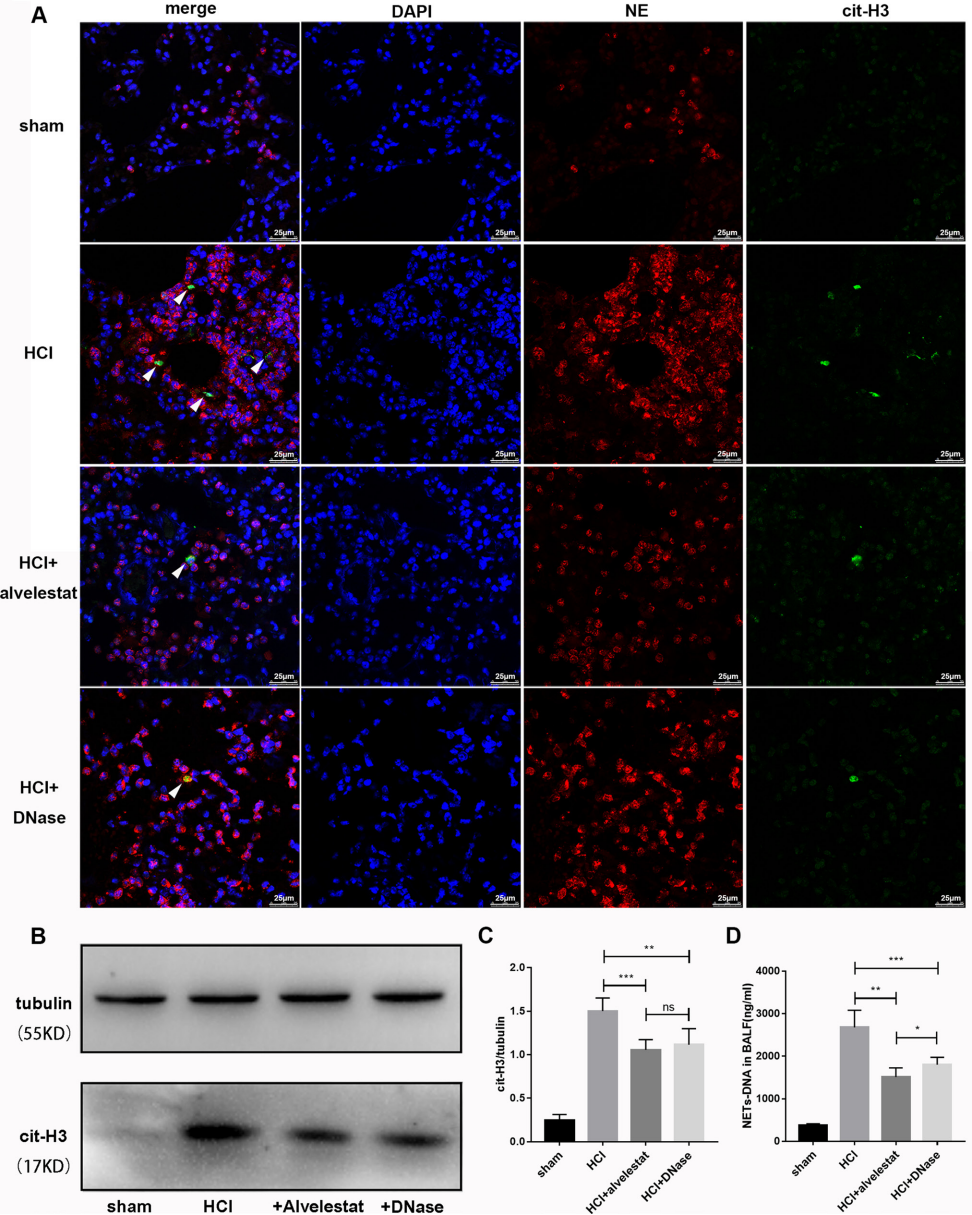
Alvelestat or DNase attenuated NET formation in aspiration-induced ARDS model (**A**) NETs were located by confocal microscopy of lung section with immunofluorescence in the sham group, HCl group, HCl plus alvelestat group and HCl plus DNase group at 12 h. Visualization of the complex of cit-H3 and NE (NETs, white arrow) was reduced in mice after administration of alvelestat and DNase. Scale bars: 50 μm. (**B**) An example of cit-H3 and tubulin detected by Western blot in the lung tissues of each group, and the cit-H3/tubulin ratio (**C**) decreased in mice with administration of alvelestat and DNase, with parallel results of NET-DNA in BALF(D). (^*^p < 0.05, ^**^p < 0.01, ^***^p < 0.001).

**Figure 5:**
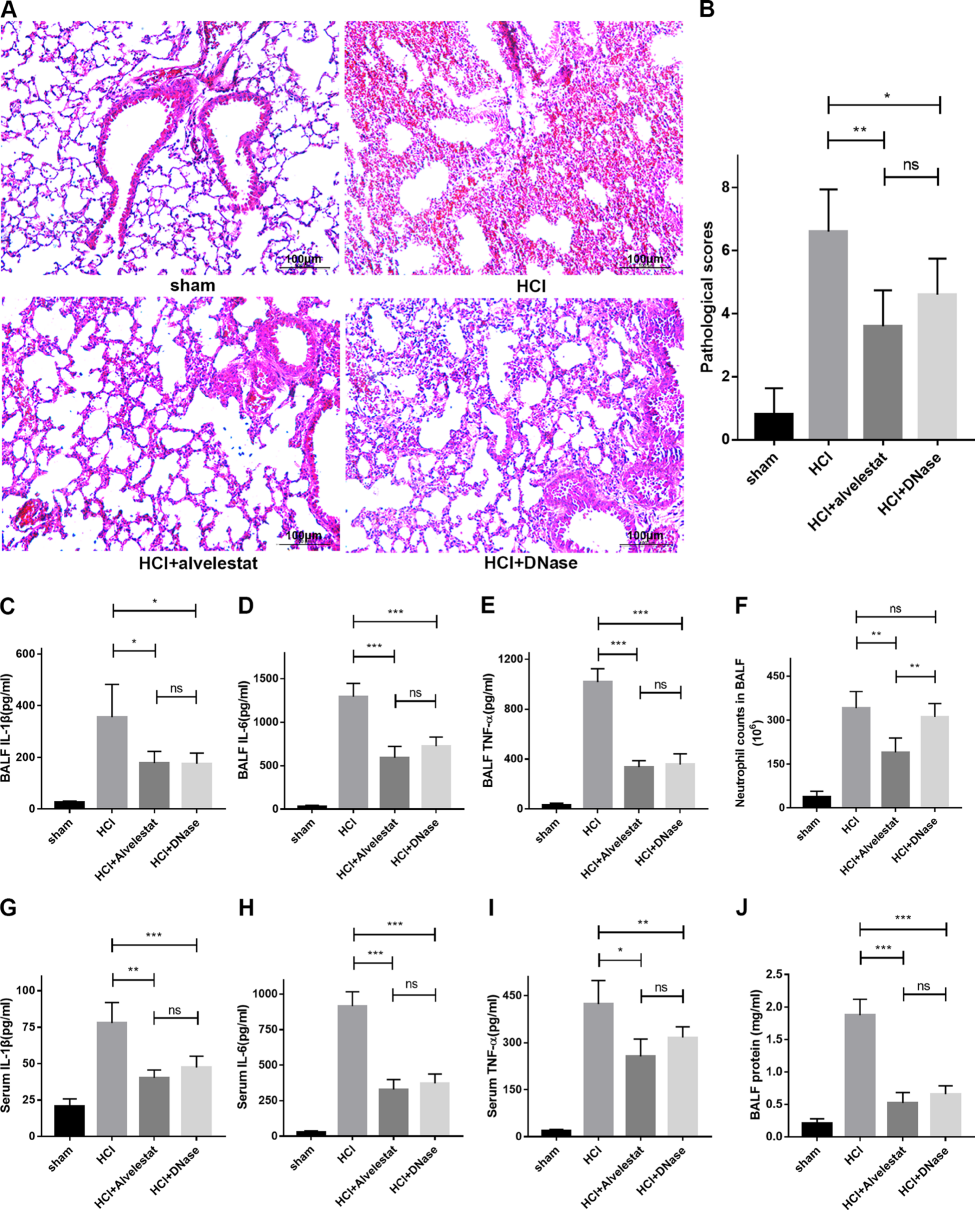
Alvelestat or DNase provided similar protection against ARDS (**A**) Administration of alvelestat or DNase significantly alleviated lung damage in HCl-treated mice. A representative image of H&E-stained sections indicates substantial improvement in the lungs of alvelestat or DNase-treated mice at 12 h. (**B**) Lung histological scores were lower in the alvelestat or DNase plus HCl groups than in the HCl group. BALF (**C**) IL-1β, (**D**) IL-6 and (**E**) TNF-α were notably decreased in the alvelestat or DNase-treated groups compared with the HCl-treated group. (**F**) There was no difference in neutrophil counts in BALF between the DNase plus HCl groups and the HCl only group, but neutrophil counts in BALF of alvelestat-treat mice were alleviated. Serum (**G**) IL-1β, (**H**) IL-6 and (**I**) TNF-α (F) were also notably decreased in the alvelestat or DNase-treated groups compared with the HCl-treated group. (**J**) Administration of alvelestat or DNase also reduced total protein in BALF of HCl-treated mice (^*^p < 0.05, ^**^p < 0.01, ^***^p < 0.001).

In this study, we also examined whether alvelestat, a neutrophil elastase that inhibits NET formation, has protective potential. DNase I, a NET degradation agent that was shown to decrease NETs *in vivo* in our previous study [9], was administered separately as a positive control. The counts of the citrullinated histone-NE complex (immunofluorescence characterization of NETs) in random fields were substantially reduced in lung tissues of the alvelestat-treated group, similar to the DNase-treated group, as shown by confocal microscopy (Figure 4A). Similar results were observed for the cit-H3/tubulin ratio (Figure 4B, 4C) in lung tissue and the NET-DNA concentration (Figure 4D) in BALF as semi-quantitative or quantitative analyses of NETs, respectively. Furthermore, both alvelestat and DNase I reduced lung injury in acid-treated mice as demonstrated by improved lung histology along with NET attenuation (Figure 5A, 5B). BALF and serum levels of IL-1β, IL-6, and TNF-α (Figure 5C–5J) were reduced following inhibition of NET production. Collectively, these findings confirmed that NETs play a role in the progression of ARDS, and targeting NETs by inhibition or degradation is protective.

### *In vitro* studies

**Figure 6:**
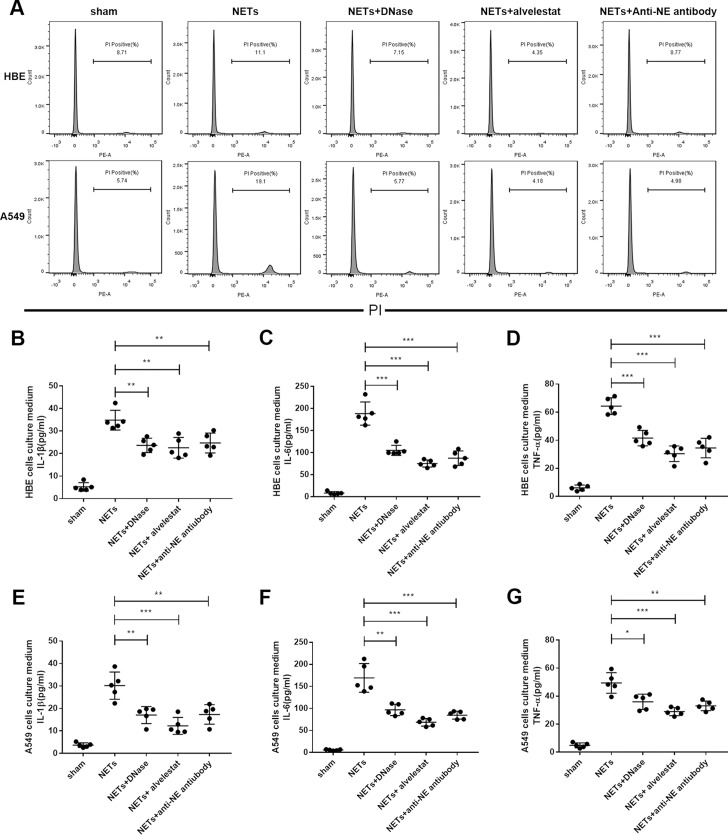
Alvelestat and DNase inhibited NET-mediated cell death *in vitro* (**A**) PIstaining of cell death via flow cytometry indicated that preincubation of exogenous NETs with anti-NE antibody or alvelestat and DNase could significantly decrease A549 cell death *in vitro*. Administration of exogenous NETs in HBE cells (**B**–**D**) and A549 cells (**E**–**G**) increased the levels of IL-1β, IL-6, and TNF-α in the supernatant of culture medium, whereas anti-NE antibody, alvelestat or DNase preincubation substantially decreased these cytokines (^*^p < 0.05, ^**^p < 0.01, ^***^p < 0.001).

As NE is a major component of NETs, we further examined whether alvelestat-mediated protection was also dependent on inhibition of NE activity in NETs. To address this issue, we first stimulated adenocarcinoma human alveolar epithelial cells (A549) and human bronchial epithelial cells (HBE) with exogenous human-derived NETs and observed that administration of NETs enhanced cell death in A549 cells, while NETs barely caused mild damage in HBE cells as shown by PI staining and flow cytometry analysis (Figure 6A). However, NETs could induce an inflammatory response in both A549 cells and HBE cells (Figure 6B), confirming that NETs possess direct cytotoxic effects and induce inflammation. Co-incubation of exogenous NETs with alvelestat, anti-NE antibody and DNase I decreased cytotoxicity and inflammatory responses, as demonstrated by the significantly alleviated cell death and reduced inflammatory cytokine levels in the supernatant of the culture medium. These results confirmed that cytoprotection and inflammatory responses are mediated by targeting NETs and their components, such as NE.

## DISCUSSION

To date, ARDS is still the leading cause of respiratory failure in critically ill patients in the ICU and has high morbidity and mortality rates [1, 2]. Gastric aspiration is one of the leading causes of ARDS [3, 23, 24]. There is a broad range of conditions that predispose individuals to gastric aspiration-induced ARDS (e.g., general anesthesia and neurologic disorders), and 30–50% of gastric contents aspiration-induced ARDS patients eventually die of respiratory failure [3] despite receiving standard therapy. However, in contrast to sepsis-associated ARDS, the underlying mechanism of acid aspiration-induced ARDS is not well-known.

Accumulating evidence demonstrates that uncontrolled and persistent inflammatory responses are deeply implicated in the progress of ALI/ARDS, which is characterized by massive leukocyte activation and cytokine release [21]. Moreover, damage-associated molecular pattern molecules (DAMPs), such as mitochondrial DNA, extracellular histones, high mobility group box-1 protein, and formyl peptides, have been shown to be a primary cause of uncontrolled inflammation in addition to pathogens [25–28]. During the process of NETosis, neutrophils (the most common type of infiltrating leukocytes) produce and release NETs, which are formed of extracellular deoxyribonucleic acid (eDNA) coated with histones, proteases and granular and cytosolic proteins, act as a danger-associated molecular patterns and are associated with inflammation and tissue injury [29]. As DAMPs, NETs also induce damage and inflammatory responses in several pathological scenarios in different organs by opposing the NETosis of neutrophils [30]. The aim of this study was to not only explore the relationship between the NET level and the disease severity of aspiration-induced ALI/ARDS but also to test the efficacy of attenuation of NETs as a treatment for ALI/ARDS in an experimental model of acid-induced lung injury.

Here, we found that the levels of NETs correlated with the disease severity of ALI/ARDS. Thus, we concluded that NETs may serve as a marker for clinically monitoring the severity of lung injury in humans. Then, we prepared a murine model of HCl aspiration-induced ARDS to study whether NETs are clinically relevant in aspiration of gastric acid and are associated with high mortality rates of ARDS [16, 21, 22]. HCl exerts initial chemical damage to pulmonary airway epithelia, which triggers an inflammatory response, followed by pulmonary edema and disruption of the alveolar capillary membrane [21, 22, 31]. We demonstrated that HCl aspiration caused severe lung injury in mice, together with significantly increased levels of NETs in BALF of mice, which were correlated with the severity of ALI/ARDS.

Large quantities of NETs in the extracellular milieu have pathological importance. NETs and their components were shown to have direct cytotoxicity in endothelial and epithelial cells [19]. Additionally, NETs can serve as a DAMPs to promote inflammation by mediating downstream inflammatory responses that lead to cytokine production [32, 33]. Consistent with these studies, we found that exogenous NETs aggravated HCl-induced ALI in mice, whereas blockade of NETs provided significant protection against ALI/ARDS. These findings thus confirmed the significant pathological and targetable role of NETs in acid-induced ALI/ARDS. Based on our results, we propose a possible role of NETs in mediating inflammation and lung injury after acid challenge. In brief, neutrophil infiltration in response to HCl challenge releases large quantities of NETs, which are directly cytotoxic or act as endogenous DAMPs to promote innate immunity and systemic inflammation. Furthermore, NETs may, in turn, attract more inflammatory cells to damaged sites and amplify inflammation by increasing cytokine production, further contributing to ALI/ARDS. Animal studies suggest that NET attenuation (such as NE inhibitors) could be a potential therapeutic strategy. Furthermore, as an oral drug, alvelestat might provide a novel pharmacological approach to treat ALI/ARDS by targeting NETs in humans.

Notably, DNase has a protective effect as a NET degradation agent *in vivo* and *in vitro* [9, 34–36]. NE plays a central role in NET generation [17], and NE is also a component of NETs. Alvelestat, an NE inhibitor, can alleviate inflammatory responses [20, 37]. In this study, we reported that alvelestat-treated and DNase-treated mice with ALI had improved survival rates and reduced lung inflammation compared to control mice. *In vitro*, A549 cells were vulnerable to NETs, while HBE cells survived with NETs, suggesting that NETs may affect different mechanisms in cell death. However, inhibition of NE with antibody and inhibition of NE activity with alvelestat or degradation of NETs with DNase all significantly alleviated the A549 cell mortality rate. Furthermore, the culture media supernatant levels of IL-1β, IL-6, and TNF-α were reduced in NE antibody-treated, alvelestat-treated and DNase-treated cells.

We thus hypothesize that inhibition of NE activity not only decreases NETs *in vivo* but also reduces the cytotoxicity or the inflammatory response of NETs to airway epithelial cells. Our findings suggest that drugs targeting NETs may be a therapeutic option in patients with ARDS.

In summary, our data showed that NETs play an inflammatory role in acid aspiration-induced ALI/ARDS by stimulating systemic inflammation and contributing to lung damage. The levels of NETs may act as a novel marker indicating disease activity in mice and humans with ALI/ARDS. Blockade of NETs by a specific inhibitor or NET degradation agents protected against ALI in mice. As expected, NETs and their components, such as NE, directly possess cytotoxic effects and induce an inflammatory response. A better understanding of the mechanisms underlying NET release induced by HCl and the effects of NETs on host immune system modulation will support the development of potential new therapeutic strategies for ALI/ARDS.

## MATERIALS AND METHODS

### Collection and preparation of patient bronchial aspirates

A total of 13 patients with ARDS caused by aspiration of gastric contents admitted to the intensive care units (ICU) of the Internal Nerve Medicine or Respiratory Internal Medicine departments of the Xiangya Hospital, Central South University, Changsha, People's Republic of China were enrolled in this study. All subjects met the criteria for ARDS defined by the Berlin definition: (1) acute onset of dyspnea with history of aspiration within one week and received incubation for mechanic ventilation support, (2) PaO_2_/FiO_2_ equal to or less than 300 mmHg over 48 h, and (3) new infiltration shadow observed on chest radiography [38]. Patients who had sepsis or ARDS for more than three days were not included. A disposable suction catheter with mucus trap (Yueyue, China) was passed through the endotracheal tube to immediately collect samples of bronchial aspirate when patients were diagnosed with ARDS. Bronchial aspirate clearance is a routine practice operation in ventilation patients, and this study was approved by the Ethics Committee of Xiangya Hospital. Collected aspirates were homogenized with 0.1% dithiothreitol (DTT), filtered, and centrifuged with 500 g at 4°C. Cell-free supernatant was stored at -80°C for NET-DNA qualification for assessment of NET levels. Aspirate smears were prepared for qualitative analysis of NETs with immunofluorescence microscopy.

### Animals and acid-aspiration-induced acute lung injury model

Six- to eight-week-old male C57BL/6 mice, weighing 18–25 g, were purchased from the Department of Experimental Animals, Central South University (Changsha, China). Mice were housed in a constant temperature at 25°C with a 12 h dark–light cycle and allowed to acclimate to the environment for three days before experimentation. All experimental protocols of this study were approved by the Institutional Animal Care and Use Committee of Health Sciences Center, Central South University, Changsha, People's Republic of China.

For the induction of acid-induced lung injury, mice were anesthetized with sodium pentobarbital (50 mg/kg) and challenged by intratracheal instillation of hydrochloric acid (HCl, 0.1N,1.5 μl/g) into the lung. The mice in the control group underwent a similar procedure but received sterile saline instead.

### Evaluation of ARDS in mice

The severity of ARDS was evaluated by blood gas analysis, pulmonary edema, alveolar epithelial permeability and lung histology. Left ventricle paracentesis was processed with 1 ml syringes to obtain arterial blood. We analyzed arterial partial oxygen tension (PaO_2_) of arterial blood with a gas analyzer at different time points after hydrochloric acid aspiration. The wet to dry lung weight ratio was calculated to assess pulmonary edema. Lung tissues were rapidly cut and flushed in phosphate-buffered saline (PBS) to remove the remaining blood. After excessive PBS was carefully removed by tissue paper, the lung tissues were weighed as wet weight. Then, the tissues were parched in an oven at 60°С for 72 h, followed by a second weighing as dry weight.

Protein levels and the number of neutrophils in BALF were measured to evaluate alveolar epithelial permeability. We used 1 ml PBS for lavage of mouse lungs three times to obtain BALF. BALF was centrifuged at 500 × g for 10 min at 4°C. Cell-free supernatant protein concentration was measured by a bicinchoninic acid protein assay (Biomiga, USA), while the pellets were resuspended for a cell suspension for neutrophil count by performing Wright's staining on a glass slide.

For lung histology, part of the lung tissues was fixed with 4% paraformaldehyde and embedded in paraffin, and 5 μm sections were acquired and stained with hematoxylin and eosin. The stained sections were evaluated and scored in random visual fields. The severity of lung injury was assessed with a semi-quantitative analysis by scoring from 0 (no lesion) to 4 (significant and extended lesions) according to the following parameters: vascular congestion, alveolar necrosis, neutrophil infiltration and macrophage infiltration

### Preparation of mouse samples

In another group of mice, 1 ml PBS was used in the lung to obtain BALF. Centrifuged cell-free supernatants were stored at −80°C until further analysis. The lung lobes were rapidly excised, flash frozen in liquid nitrogen, and stored at −80°C. Mouse blood was collected by retro-orbital bleeding into a tube containing EDTA as an anticoagulant and centrifuged to separate plasma, and the plasma was stored at −80°C.

### Generation, isolation and purification of NETs

Mouse neutrophils were isolated from bone marrow and purified by density gradient centrifugation as previous described [39]. Briefly, the muscles and joints from the femur and tibia, which were collected from euthanized mice, were removed without breaking the bones. Then, epiphyses of the bones were cut, and bone marrow was flushed with a 1 ml syringe at both ends of the bone. Next, cells were suspended in a volume of 3 ml, followed by red blood cell lysis. Then, 3 ml of Histopaque 1119 was added in a 15 ml tube at the bottom with 3 ml of Histopaque 1083 overlaid, and the bone marrow cell suspension was added on the top. The layered solution was centrifuged without a brake, and the neutrophils at the interface of the Histopaque 1119 and Histopaque 1083 layers were collected with neutrophils typically > 95% viable and > 90% pure.

Human neutrophils were isolated from peripheral blood donated by healthy volunteers as previously described [40]. Briefly, blood was overlaid on lymphocyte separation solution (LSM), and the layered solution was centrifuged at 800 g for 30 min at 21°C without a break. The bottom red layer was retained and resuspended with 3% Dextran-PBS solution; then, the solution was incubated for 30 min. Next, the supernatant was transferred into fresh tubes for centrifugation for red blood cell lysis and neutrophil enrichment, with neutrophils typically > 95% viable and > 95% pure.

Both mouse and human-derived neutrophils were stimulated with 500 nM of PMA for four h at 37°C and 5% CO_2_ to allow NETosis. The media were gently aspirated and discarded, while the bottom of the dishes was washed with cold PBS to remove all adherent material. The solution was centrifuged for 5 min at 450 g at 4°C to remain cell-free NET-rich supernatant. The supernatant continued to spin for 10 min at 18,000 g at 4°C to allow the DNA to pellet. The pellets were all obtained in PBS at a concentration corresponding to 2 × 10^7^ neutrophils per 100 μl. The cell-free NET stock yielded a DNA concentration range between 140–180 ng/μl measured by a DNA quantification tool.

### Treatment with exogenous NETs, alvelestat and DNase

In a separate set of experiments, we examined whether NETs released after acid-caused lung injury contributed to the pathogenesis of ARDS. To this end, mouse-derived NETs with a DNA concentration of 1.5 mg/kg were administered to mice by intratracheal injection shortly after the HCl challenge. Meanwhile, another group of mice received a(5 mg/kg) by subcutaneous injection immediately after acid aspiration, and some received DNase I (5 mg/kg) as a positive control, with the aim of further confirming the pathogenic role of NETs as well as exploring possible treatment strategies. The dosages of NETs, alvelestat and DNase were based on previous experiments [9, 20, 41].

### Qualitative and quantitative analysis of NETs

NETs detected in sputum of patients’ bronchial aspirates and mouse lung tissues we identified by co-localization of cit-H3 and NE by immunofluorescence staining. Briefly, sputum smears were prepared and fixed in PLP fixative (3% paraformaldehyde, L-lysine monohydrate, sodium m-periodate, 0.1 M disodium hydrogen orthophosphate), dried and coated in 15% sucrose. For cit-H3 and NE detection, slides were blocked with PBS containing 2% BSA and 3% donkey serum and then incubated for 2 h at room temperature (RT) with rabbit anti-cit H3 antibody (Abcam) and goat anti-NE antibody (Santa Cruz) as primary antibodies. Paraffin-embedded mouse lungs were sectioned (5 μm) and mounted on glass slides. After dewaxing, permeabilizing and blocking, the sections were incubated with rabbit anti-cit-H3 antibody (Abcam) and goat anti-NE antibody (Santa Cruz, sc-9520) overnight at 4°C.All samples were detected with Alexa Fluor 488 donkey anti-rabbit (Abcam) and Alexa Fluor 647 donkey anti-goat (1:500; Abcam) secondary antibodies for 1 h at RT and mounted using ProLong Gold Antifade Mountant with DAPI (Life Technologies, Carlsbad, CA, USA) to detect DNA.

For determination of NET levels in cell-free bronchial aspiration supernatants of patients and the mouse BALF, extracellular DNA was quantitated using the Quant-iT PicoGreen dsDNA Assay Kit (P7589, Invitrogen, Carlsbad, CA) followed the manufacturer's instructions. This assay selectively detects double-stranded DNA, which we quantitated in 10 μL of bronchial aspiration supernatant and mouse BALF against a DNA standard curve of 0–2000 ng/μL.

Western blot assays were also performed using BALF and whole cell lysates obtained from mouse lung tissues as a semi-quantitative analysis of NETs for cit-H3, which can be a specific predictor of NETs. Membranes were incubated overnight with the anti-Cit-H3 (1:1000, Abcam) antibody and an anti-GAPDH antibody (1:2000, Proteintech) as an internal control.

### Quantification of inflammatory indicators

IL-1β, IL-6 and TNF-α levels in patients’ bronchial aspiration supernatant and mouse plasma and BALF were determined using commercially available human and mouse IL-1β, IL-6 and TNF-α enzyme-linked immunosorbent assay (ELISA) kits (eBioscience, USA) according to the manufacturer's instructions.

### Assay for NET cytotoxicity *in vitro*

Human bronchial epithelial cells (HBE) and human alveolar epithelial cells (A549) were obtained from the American Type Culture Collection. Cells were cultured in Dulbecco's modified Eagle's medium (DMEM) supplemented with 10% fetal bovine serum and 1% penicillin-streptomycin. After the cells had grown to 70–80% confluence, they were stimulated with human-derived NETs (DNA concentration at 5 μg/ml) for 16 h at 37°С. In another set of experiment, cells added to NETs were cocultured with anti-NE antibody (20 μg/ml) or alvelestat (20 μg/ml). Cells were collected and stained with propidium iodide to analyze cell damage via flow cytometry. Additionally, cell culture supernatants were analyzed for IL-1β, IL-6, and TNF-α levels with ELISA kits from eBioscience (eBioscience, USA), according to the manufacturer's protocol.

### Statistical analysis

Results are expressed as the mean ± standard deviation(SD). Differences among more than two sets of data were assessed by performing one-way ANOVA followed by Tukey's multiple comparisons test. A value of *P* < 0.05 (two-tailed) was considered statistically significant.
